# Eating while intoxicated: characterizing the molecular mechanism behind *V. cholerae* toxin MakA-regulated autophagy

**DOI:** 10.1080/15548627.2022.2146893

**Published:** 2022-11-21

**Authors:** Dale P. Corkery, Yao-Wen Wu

**Affiliations:** a Department of Chemistry, Umeå University, Umeå, Sweden; b Umeå Centre for Microbial Research, Umeå University, Umeå, Sweden

**Keywords:** Cholesterol, MakA, non-canonical autophagy, pore-forming toxin, *Vibrio cholerae*

## Abstract

Extracellular pathogens utilize secreted virulence factors to regulate host cell function. Recently we characterized the molecular mechanism behind host macroautophagy/autophagy regulation by the *Vibrio cholerae* toxin MakA. Cholesterol binding at the plasma membrane induces MakA endocytosis and pH-dependent pore assembly. Membrane perforation of late endosomal membranes induces cellular membrane repair pathways and V-ATPase-dependent unconventional LC3 lipidation on damaged membranes.

Autophagy plays an essential role during pathogen infection as a regulator of both innate and adaptive immune responses. Beyond its well-established role as a cell-autonomous defense system against intracellular pathogens (xenophagy), the autophagic machinery has been implicated in a growing list of immune-modulating processes including cytokine secretion, antigen presentation and general maintenance of immune cell homeostasis/function. As a consequence, many pathogens have evolved sophisticated strategies to engage and subvert host autophagy responses.

Recently, we characterized the molecular mechanism behind host autophagy regulation in cells exposed to the *Vibrio cholerae* toxin MakA [1]. Using liposome sedimentation and flotation assays and giant unilamellar vesicles/GUVs, we identified cholesterol as the primary lipid responsible for interaction between MakA and membranes. This interaction is pH-dependent with the highest binding observed between pH 5.4 and 6.8 *in vitro*. It is worth noting that the small intestine, the preferential site of colonization for *V. cholerae*, maintains a pH gradient ranging from 6.2 to 7.9. This suggests that pH-dependent cholesterol binding could serve as a mechanism to restrict MakA intoxication to a defined site within the host.

MakA is expressed from an operon which encodes two additional toxins, MakB and MakE, both of which have a high degree of structural similarity to MakA. However, neither MakB nor MakE bind to membranes containing cholesterol allowing us to take advantage of slight differences in hydrophobic residues between MakA and the MakB and MakE toxins to map the potential cholesterol interacting region of MakA. We identified an isoleucine pair (I236 and I237) in the β3-α5 loop of MakA that is essential for its interaction with cholesterol. This is unlike the conserved cholesterol binding motif (Thr-Leu pair) of cholesterol-dependent cytolysins/CDCs and could represent a novel cholesterol-binding mechanism. Interestingly, the cholesterol-binding deficient mutant (MakA^I236D,I237D^) exists exclusively in monomeric form, while 20% of wild-type MakA exists as an oligomer in solution, as shown by size exclusion chromatography. This suggests that MakA oligomerization can be regulated by cholesterol interaction in a pH-dependent manner.

To observe the state of membrane-bound MakA under low pH, monomeric MakA was incubated with nanodiscs (a synthetic model membrane system) at pH 5.4 and imaged using cryo-EM. Under these conditions, MakA assembles into propeller-like oligomeric structures with an ~3-nm pore in the center. Liposome leakage assays were used to confirm the pH-dependent membrane-perforating activity of MakA.

Translating these findings into the cell, we observed recruitment of the Endosomal Sorting Complex Required for Transport (ESCRT) machinery to MakA-containing endolysosomal membrane aggregates, consistent with activation of the ESCRT-mediated membrane repair pathway in response to MakA pore formation. Importantly, LGALS/galectin proteins are not recruited to damaged membranes, suggesting membrane lesions induced by MakA are not extensive enough to induce the autophagy-dependent membrane removal pathway. This finding is consistent with the small pore size observed *in* *vitro*.

Endosomal maturation is accompanied by a decrease in pH from 6.5 in early endosomes to 5.5 in late endosomes, mediated by the proton pumping ability of V-ATPase. We hypothesized that this gradual decrease in pH can regulate MakA pore formation, restricting pore formation to acidified late endosomal membranes. In agreement, inhibition of endosomal acidification with the V-ATPase inhibitor bafilomycin A_1_ prevents accumulation of damaged endolysosomal membranes in MakA-treated cells. However, expression of the *Salmonella* T3SS effector SopF, which inhibits ATG16L1 recruitment to the V-ATPase without inhibiting endosomal acidification, prevents LC3 lipidation, but does not prevent MakA-induced accumulation of damaged endolysosomal membranes in cells. Therefore, MakA-induced endolysosomal damage signals through the V-ATPase-ATG16L1 axis to induce unconventional LC3 lipidation similar to what has been reported for the pore-forming toxin listeriolysin O/LLO from *Listeria monocytogenes*, which induces unconventional LC3 lipidation by listeriolysin O-inflicted damage to the phagosomal membrane. The presence of LC3 on these damaged membranes raises the intriguing question of what function unconventionally lipidated LC3 serves on damaged endolysosomal membranes, if not exclusively for LGALS-dependent membrane removal.

Collectively, our findings reveal the molecular mechanism behind MakA-regulated autophagy (Figure 1). By coupling pore formation to pH, MakA-induced membrane damage is limited to the late endosomal compartment, avoiding cytotoxicity that can be associated with plasma membrane damage. It also raises the possibility of recurrent membrane damage whereby MakA pore assembly in the late endosome perforates the membrane causing an increase in pH and pore closure/disassembly. Successful repair of the membrane leads to re-acidification of the endosome and re-assembly of the pore thereby creating a state of chronic membrane damage and repair. How such a state would benefit the extracellular pathogen remains unknown. We hypothesize that chronic activation of the noncanonical autophagic pathway at damaged membranes could dampen other autophagy-related pathways utilizing the same machinery (i.e., antigen presentation and cytokine secretion). Future studies examining the immunosuppressive function of MakA *in vivo* will help shed light on the link between chronic autophagy regulation and extracellular pathogen fitness.

**Figure 1.**
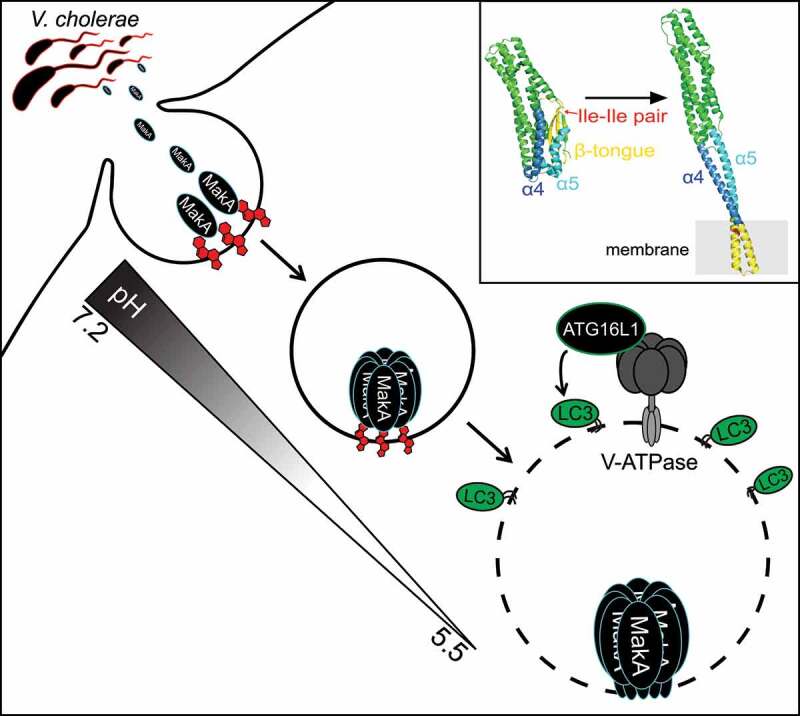
Model of MakA-induced autophagy. Interaction between MakA and cholesterol at the plasma membrane facilitates MakA endocytosis. Endosomal acidification promotes pore-assembly resulting in V-ATPase-dependent unconventional LC3 lipidation on the damaged membrane. Upper right box shows structures of MakA monomer (PDB 6EZV) and a subunit of the MakA pore from the tubular model (PDB 7P3R). The regions that undergo conformational changes are highlighted: β-tongue (yellow), α4 (blue) and α5 (light blue). The cholesterol-interacting Ile-Ile pair is shown in red.
